# Nigromyces azzae gen. nov., sp. nov.: a novel black yeast isolated from a mangrove tree in Kuwait

**DOI:** 10.1099/ijsem.0.007128

**Published:** 2026-04-10

**Authors:** Haya Al-Sammar, Teun Boekhout, Nouf Al-Mutairi, Sheikha Al-Zarban

**Affiliations:** 1 Department of Biological Sciences, Faculty of Science, Kuwait University, Kuwait; 2 College of Science, King Saud University, Riyadh, Saudi Arabia; 3 The Yeasts Foundation, Amsterdam, Netherlands

**Keywords:** black yeasts, mangroves, *Zalariaceae*

## Abstract

As part of a preliminary investigation of yeast diversity associated with grey mangrove trees (*Avicennia marina*) in Kuwait, a total of 17 yeast isolates were recovered from leaves and soil of trees in Shuwaikh. Among them, one strain (Shy33) exhibited distinct morphological and molecular characteristics that could not be assigned to any known species and was therefore described as a novel taxon, *Nigromyces azzae* gen. nov., sp. nov. Phylogenetic analyses based on the internal transcribed spacer (ITS1–5.8S–ITS2), SSU rDNA and LSU rDNA regions and partial *β*-tubulin gene (*TUB2*) revealed that the strain is closely related to members of the genus *Zalaria* but forms a distinct lineage within the family *Zalariaceae* (order *Dothideales*). The strain exhibited typical black yeast-like features, including melanized cell walls, meristematic growth and budding yeast cells. Physiological testing showed that the strain lacked fermentative capacity but could assimilate a variety of carbon sources such as d-glucose, sucrose, cellobiose, salicin and d-mannitol. The strain demonstrated growth at temperatures between 21°C and 42°C but not at 18°C or 45°C. Based on these findings, we propose a new genus and species, *N. azzae* gen. nov., sp. nov., with Shy33 as the type strain (CBS 148914^T^ = MUCL 58501^T^).

**Impact statement**

In this study, we present the discovery and taxonomic characterization of *Nigromyces azzae*, a novel black yeast, isolated from the mangrove tree *Avicennia marina* in Kuwait. Phylogenetic analyses based on multilocus sequence data confirmed that the strains represented a distinct lineage within the family *Zalariaceae*, justifying the proposal of a new genus and species. The identification of this novel taxon expands current knowledge on the relatively recently described family *Zalariaceae*. Furthermore, this study enhances our understanding of the biodiversity of black yeast-like fungi and yeasts in the underexplored mangrove ecosystems of the Arabian Gulf, particularly in Kuwait. Although based on a single strain, this finding highlights the potential for mangrove habitats of the Arabian Gulf to harbour previously uncharacterized fungal taxa and underscores the need for broader ecological surveys of black yeasts in the region.

## Data summary

The authors confirm that all supporting data, code and protocols have been provided within the article or through supplementary data files.

## Introduction

Black yeasts are a group of dematiaceous fungi that exhibit filamentous asexual morphology and budding yeast-like growth during their life cycle. Many species display meristematic growth, characterized by slow extension and the formation of aggregated melanined cells that appear as densely clumped colonies. Some members are referred to as microcolonial fungi owing to their limited expansion on or within natural substrates such as rocks [1,2]. In addition to their pleomorphic nature, black yeasts represent a taxonomically diverse and polyphyletic group with most species classified within the order *Dothideales* or *Chaetothyriales* [1].

Black yeast communities are common inhabitants of extreme environments exposed to UV radiation, high osmotic pressure, elevated temperatures and oligotrophic conditions [2]. Their morphological plasticity and distinctive adaptive strategies enable them to survive under conditions that are inhospitable to many other microorganisms [2,3]. Owing to their remarkable survival capabilities, some species are mostly confined to extreme environments and are referred to as specialists [4]. For example, *Hortaea werneckii*, which is frequently isolated from hypersaline and saline environments, can grow under extremely high salt concentrations, such as those found in solar salterns, and in the absence of salt [5,7]. Other black yeasts are known to be polyextremotolerant generalists and are capable of colonizing a wide range of stressful environments [4,8]. *Aureobasidium pullulans*, for instance, is a well-known polyextremotolerant generalist found in diverse habitats, including indoor environments, plant surfaces, glaciers and hypersaline ecosystems [6,9,11].

Given the distinctive adaptability of black yeasts and other extremotolerant fungi, their presence is not limited to terrestrial and hypersaline environments but extends to coastal ecosystems such as mangroves [12,13]. Mangroves are coastal intertidal ecosystems dominated by facultative halophytic trees and shrubs that thrive in tropical and subtropical regions. These ecosystems provide critical ecological functions such as shoreline protection, carbon sequestration and support for diverse marine and terrestrial life. Mangroves are distributed across several countries in the Arabian Gulf and collectively cover ~165 km². Despite the region’s extreme climatic conditions, characterized by high salinity, limited freshwater input and high environmental temperature, mangrove stands are primarily dominated by *Avicennia marina* trees. This species is found on the coasts of the United Arab Emirates (UAE), Saudi Arabia, Qatar, Bahrain, Kuwait and southern Iran. Iran and the UAE host the largest mangrove areas in the region, covering ~78.76 and 65.24 km², respectively [14].

In Kuwait, natural mangrove habitats, although historically thriving, are rare due to extreme temperature variations and severe dry winds [14,15]. Recognizing their ecological significance in mitigating climate change, improving water quality and supporting marine biodiversity, *A. marina* propagules were reintroduced along Kuwait’s protected embayments, namely, Kuwait Bay, Sulaibikhat Bay, Shuwaikh and in the Subbiya channel along the shore of Bubiyan Island [16,17]. These mangrove plantations not only enhance fisheries and marine productivity but also serve as potential reservoirs for diverse microbial communities, including yeast and filamentous fungi [18]. Fluctuating salinity, high temperatures and tidal influences in these habitats create an environment that favours salt-tolerant fungi, similar to those found in hypersaline ecosystems [19].

As part of a large-scale study on yeast diversity in the mangrove ecosystems of Kuwait, a preliminary investigation was conducted in which mangrove trees in the Shuwaikh area were sampled, resulting in the isolation of 17 yeast strains. Most of these isolates were identified to the species level based on sequencing of the D1/D2 domain of 26S rDNA. However, one strain (Shy33) exhibited unique morphological features characteristic of black yeast-like fungi and could not be assigned to any known species. Multilocus sequence analysis revealed that Shy33 represents a genetically distinct lineage, clearly separated from its closest relatives in the genus *Zalaria*. Based on its unique phylogenetic placement and distinctive morphological traits, we propose the name *Nigromyces azzae* gen. nov., sp. nov. for this novel taxon.

## Methods

### Isolation of the yeasts

Fresh leaves, dry leaves and soil were collected from four *A. marina* trees in a mangrove site in Shuwaikh, north of Kuwait (29° 20′ 02.0″ N 47° 53′ 58.4″ E) during September 2021. Samples were collected for a preliminary investigation of the yeast community in the mangrove trees of Kuwait. The trees were spaced 1.5–6.0 m apart, and the samples were obtained from three distinct regions within each tree, in which each location was 1.0–1.5 m apart. All samples were placed in sterile plastic bags and transported to the laboratory in an icebox to maintain sample integrity.

The media used for the isolation of yeasts were Sabouraud Dextrose Agar (SDA; Oxoid, UK), yeast extract peptone dextrose agar (YPDA) medium containing 1% (w/v) yeast extract, 2% (w/v) Bacto-peptone, 2% (w/v) glucose and 2% (w/v) agar and a modified Wickerham’s yeast–malt (YMPG) medium. The YMPG medium was composed of 0.3% (w/v) yeast extract, 0.3% (w/v) malt extract, 0.5% (w/v) peptone, 1% (w/v) glucose and 2% (w/v) agar prepared in sterile seawater to mimic the saline conditions of the mangrove habitat [20]. All media were supplemented with 200 mg l^−1^ chloramphenicol to inhibit bacterial growth.

Fresh and dry leaf samples were cut into four sections (~1–1.5 cm in size) using ethanol-sterilized scissors and placed in duplicates on each of the three isolation media. Soil samples collected from the area surrounding each tree were subjected to tenfold serial dilution, and 100 µl of the diluted suspension was spread onto the media in duplicate using sterile glass beads (~5 mm in diameter; Sigma, USA). The inoculated plates were incubated at both 30°C and 20°C, and yeast growth was monitored regularly. Yeast colonies were counted, and representative colonies were subcultured on SDA for further characterization.

### DNA extraction, PCR and sequencing

Genomic DNA was extracted from overnight yeast cultures using either the Quick-DNA Fungal/Bacterial Miniprep Kit (Zymo Research, USA) according to the manufacturer’s protocol or via a rapid boiling method. For the latter, yeast colonies were suspended in 50 µl of molecular-grade water in 0.2 ml PCR tubes. The suspensions were then heated at 95°C for 10 min in a thermal cycler (Applied Biosystems, USA). After heat treatment, the tubes were cooled on ice and centrifuged briefly, and 5 µl of the resulting supernatant was used directly as the DNA template for PCR.

Yeast isolates were identified by amplifying and sequencing the D1/D2 domains of the 26S rDNA gene using primers NL1 and NL4. For further genetic characterization of the novel species, additional loci were targeted: the internal transcribed spacer region (ITS1–5.8S–ITS2) using primers ITS1 and ITS4 [21], 28S LSU rDNA using primers LR0R and LR5 [22], SSU rDNA using primers NS1 and NS4 [21] and partial *β*-tubulin gene (*TUB2*) using primers Bt2a and Bt2b [23]. PCR was performed in a 25 µl total volume containing 4 µl of FIREPol® 5×Master Mix (Solis BioDyne), 1 µl of each forward and reverse primer (10 µM), 1 µl of genomic DNA (extracted via kit) and molecular-grade water to the final volume. Amplification was carried out using a Veriti™ 96-Well Thermal Cycler (Applied Biosystems). PCR products were visualized on 0.8% (w/v) agarose gels, and successfully amplified fragments were purified using the QIAquick® PCR Purification Kit (Qiagen, Germany). Purified PCR fragments were sequenced using the BigDye Terminator v3.1 Cycle Sequencing Kit and subsequently purified using the BigDye XTerminator Purification Kit (Applied Biosystems) according to the manufacturer’s instructions. Sanger sequencing was conducted using an ABI 3500xL Genetic Analyzer, and the results were analysed using Sequencing Analysis Software 6 (Applied Biosystems).

Raw DNA sequences were edited manually, and consensus sequences were obtained using mega v10 and BioEdit v7.2.5 [24,25]. The sequences were compared pairwise to sequences in NCBI GenBank using blast. The isolated strains were identified based on the sequences of the D1/D2 domains of LSU rDNA, with 100% similarity to reference sequences in the NCBI GenBank. Sequences of the analysed DNA loci were submitted to the NCBI GenBank database under the accession numbers PV018293–PV018308 and OR673877 for the D1/D2 domain of LSU rDNA of the isolated yeast community. The accession numbers for *N. azzae* CBS 148914^T^ are OR648401 for the ITS region, OR672588 for the SSU rDNA, OR673877 for the LSU rDNA and PQ782245 for the *TUB2* gene.

### Phylogenetic analysis

For phylogenetic analysis, sequences of the ITS region, SSU rDNA, LSU rDNA and *TUB2* gene from the putative novel species were compared to reference sequences of closely related taxa from the orders *Dothideales*, *Myriangiales* and *Endosporiales* [26]. Maximum likelihood (ML) phylogenetic analyses were conducted for individual gene datasets and for the concatenated ITS, SSU rDNA and LSU rDNA sequences. Model selection was performed using RaxmlGUI 2.0 [27], which identified GTR+I+G4 as the best-fit nucleotide substitution model based on AIC and AICc criteria. ML analyses were carried out in RaxmlGUI 2.0 [27] under the GTR+I+G4 model, and node support was assessed using 1,000 rapid bootstrap replicates. *Nothophaeocryptopus gaeumannii* and *H. werneckii* were used as outgroup taxa. Resulting ML trees were visualized in mega v10.

Bayesian phylogenetic analysis was performed in MrBayes v3.2.7a using the same substitution model as applied in the ML analysis. Two independent runs of four Metropolis-coupled Markov Chain Monte Carlo chains were performed for 10 million generations, with trees sampled every 1,000th generation. The analyses were configured to stop automatically once convergence was reached, and a 25% burn-in was applied to the initial set of sampled trees. The remaining trees were combined to generate a 50% majority-rule consensus tree and to estimate Bayesian posterior probabilities (BPPs) for all resolved clades.

### Phenotypic characterization

The morphological, physiological and biochemical characteristics of the yeast cultures were examined according to the standard methods used in yeast taxonomy [28,29]. Carbon assimilation tests were performed in duplicate in liquid media, and growth was evaluated after 7, 14 and 21 days of incubation. Nitrogen assimilation tests were carried out following an auxanographic method as described by Kurtzman *et al*. [30]. Temperature tolerance was assessed by culturing the strains on YM agar and incubating at 18°C, 21°C, 25°C, 30°C, 35°C, 37°C, 40°C, 42°C and 45°C. Microscopic images of the novel strain grown on different media were captured using a Zeiss Axioscope at magnification 100× after 10 to 2-week incubation at 25°C.

## Results and discussion

### Isolation of yeasts and yeast-like fungi from *A. marina*

A total of 17 yeasts were isolated from the fresh leaves, dry leaves and soil of the four *A. marina* trees, incubated at 20°C and 30°C on the three media (Table 1). Overgrowth of moulds was observed in these samples (data not shown), which may have restricted yeast growth. Most of the yeasts were identified to the species level based on the sequences of the D1/D2 domains of the LSU rDNA. However, strain Shy32 was identified at the genus level, in which the D1/D2 LSU rDNA sequence was identical to that of *Tortispora* sp. strain DMKU-JED5-34 (NCBI accession no. MW444868). In addition, strain Shy33 showed 96.49% similarity with *Aureobasidium* sp. strain ZY (NCBI accession no. KU051541).

**Table 1.** Identity of yeasts isolated from *A. marina* substrates at different temperatures and media based on sequences of the D1/D2 domain of the 26S rDNA

Strain code	Sample	Isolation temp. (°C)	Isolation media	Closest known species in NCBI	Query length (bp)	Identity%	NCBI accession no.
**Shy1**	Soil	30	SDA	*Candida orthopsilosis*	440	100	PV018293
**Shy6**	Dry leaves	30	YMPGA+SW	*Naganishia albida*	466	100	PV018294
**Shy11**	Leaves	20	YMPGA+SW	*Filobasidium chernovii*	302	100	PV018295
**Shy12**	Leaves	20	YMPGA+SW	*Naganishia albida*	530	100	PV018296
**Shy13**	Leaves	20	YPDA	*Filobasidium magnum*	430	100	PV018297
**Shy14**	Leaves	20	SDA	*Naganishia albida*	523	100	PV018298
**Shy15**	Soil	30	SDA	*Wickerhamiella infanticola*	432	100	PV018299
**Shy16**	Dry leaves	20	YMPGA+SW	*Naganishia albida*	515	100	PV018300
**Shy17**	Dry leaves	20	SDA	*Sporidiobolus pararoseus*	523	100	PV018301
**Shy18**	Dry leaves	20	YPDA	*Naganishia albida*	515	100	PV018302
**Shy19**	Dry leaves	20	SDA	*Naganishia albida*	450	100	PV018303
**Shy20**	Dry leaves	20	YPDA	*Naganishia albida*	530	100	PV018304
**Shy21**	Dry leaves	20	YMPGA+SW	*Naganishia albida*	516	100	PV018305
**Shy22**	Dry leaves	20	YMPGA+SW	*Naganishia uzbekistanensis*	449	100	PV018306
**Shy31**	Leaves	20	YPDA	*Candida orthopsilosis*	448	100	PV018307
**Shy32**	Soil	20	SDA	*Tortispora* sp.	451	100	PV018308
**Shy33**	Dry leaves	20	SDA	*Aureobasidium* sp.	598	96.49	OR673877

YMPGA + SW, yeast malt peptone agar prepared with seawater.

Most of the yeasts were isolated from dry leaves at 20°C, and over half of the strains were represented by the genus *Naganishia*, with *Naganishia albida* being the predominant species. *Naganishia* spp. are common inhabitants of marine environments [31,32] and can tolerate hypersaline waters [33,34]. Moreover, *N. albida* has been recorded as a part of the yeast community in mangrove forests in Thailand [13].

Two strains of the opportunistic pathogen *Candida orthopsilosis* were isolated from mangrove soil and leaves (Table 1). *Candida* species are frequently isolated from diverse marine environments including seawater, sea sediments, marine algae, mangroves, hypersaline waters and marine fish [20,33, 35]. In Kuwait, *Candida* and other yeast species have primarily been isolated from coastal seawater collected from seven distinct regions spanning the northern and southern coasts. *Candida* species accounted for 33.7% of the total yeast community, with *Nakaseomyces glabratus* (previously known as *Candida glabrata*) being the dominant species, whereas *C. orthopsilosis* represented 10% of the *Candida* strains [36]. Despite its clinical relevance, recent studies have proposed an environmental origin for some opportunistic *Candida* species, suggesting that thermotolerance may evolve in response to human-induced global warming [37,38]. This theory has been further supported by the identification of a marine *C. orthopsilosis* strain as a previously unrecognized parent of clinical hybrids, highlighting the potential role of the marine environment as both a reservoir and evolutionary driver for emerging pathogenic yeasts [39].

The black yeast community was represented by strain Shy33, which was recovered from a dry mangrove leaf cultured on SDA and incubated at 20°C (Table 1). Initial identification based on the D1/D2 domain of LSU rDNA revealed a 96.49% similarity with *Aureobasidium* sp. Given the relatively low sequence similarity for species-level identification, further molecular analyses were conducted to clarify the taxonomic placement of the strain.

### Phylogenetic analysis

The isolated yeasts were initially identified based on the D1/D2 domain of LSU rDNA (Table 1). Most strains showed 100% sequence identity to the reference strains in the NCBI GenBank database. However, the sequence of strain Shy33 (*N. azzae*) exhibited 96.5% similarity to *Aureobasidium* strain XJZ (NCBI accession no. KX263043), which differed by 18 bp. Further analysis of the ITS region revealed an 89.45% similarity between the putative new species and *Pseudohormonema sordidus* CBS 130468. Based on the sequences of SSU and LSU rDNA, *N. azzae* was found to be closely related to *Zalaria*, a genus of two species isolated from house dust, as described in 2017 [40]. The SSU rDNA sequence showed 99% similarity (9 bp difference) to *Z. obscura* DAOM 250849, whereas the LSU rDNA exhibited 95.32% similarity (40 bp difference) to *Zalaria alba* DAOM 250847. NCBI blast searches indicated that *N. azzae* belongs to the order *Dothideales*.

**Fig. 1.**
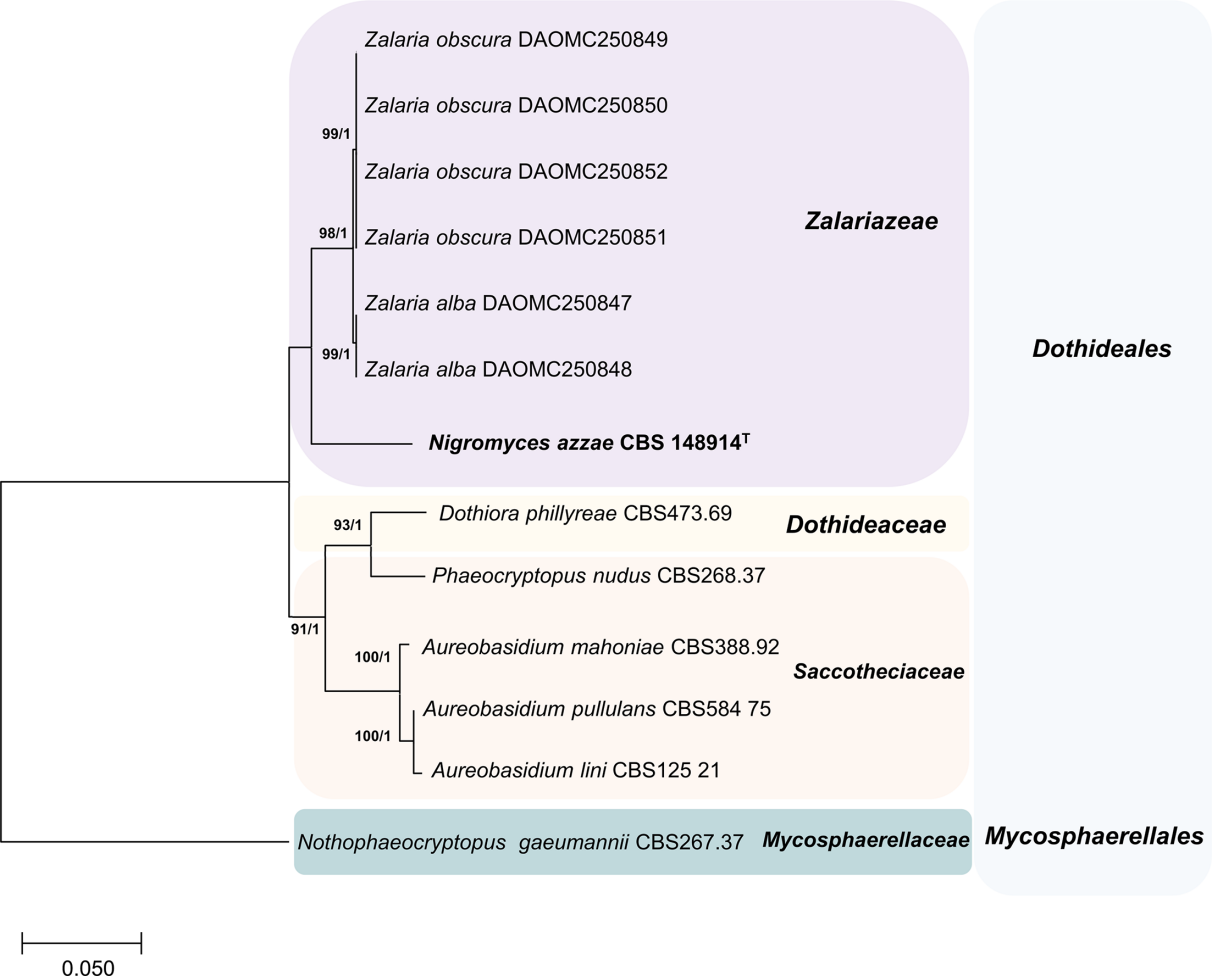
ML phylogenetic analysis based on the ITS region, 18S and 28S rRNA of *N. azzae* sp. nov. and closely related species of the closely related taxa of the *Dothideomycetes*. Species names are followed by their culture collection strain numbers. Numbers at the nodes represent ML bootstrap support values (BS ≥70%, 1,000 replicates) followed by BPP values (≥95). A dash (‘–’) indicates values below the threshold. *N. gaeumannii* was included as outgroup taxa. The scale bar indicates the number of nucleotide substitutions per site.

To further resolve *N. azzae* phylogenetic placement, analyses were conducted on individual and concatenated sequences of ITS, SSU rDNA, LSU rDNA and *TUB2* gene in comparison with closely related species from orders within *Dothideales*, *Myriangiales* and *Endosporiales* (Figs S1–S4, available in the online Supplementary Material). Phylogenetic trees constructed from these loci consistently placed *N. azzae* within the monophyletic family *Zalariaceae*, but they were distinctly separated from *Zalaria* species (Figs S1–S4). ML analysis based on the ITS region resolved the Kuwaiti strain as a distinct lineage sister to *Zalaria* spp., forming a moderately supported clade (ML=76%, BPP=1.00) (Fig. S1). This discrimination is attributable to the high sequence variability of the ITS1 and ITS2 subregions [41,42]. Similarly, individual analyses of the SSU rDNA and LSU rDNA regions resulted in the separation of *N. azzae* as a distinct lineage (Figs S2–S3). *TUB2*, in combination with other loci, is commonly used as a marker to determine the phylogenetic relationships between fungal species [43,44]. Thus, the partial sequence of *TUB2* was used to distinguish *N. azzae* from *Zalaria. N. azzae* formed a distinct clade within the phylogenetic tree based on *TUB2* sequences and was closely related to members of the family *Zalariaceae* (Fig. S4). Phylogenetic analysis based on the concatenated sequences of the ITS region, SSU rDNA and LSU rDNA confirmed the evolutionary affiliation of the novel species within the *Zalariaceae* clade as a distinct genus (Fig. 1). Thus, based on the phylogenetic analysis of individual loci and multilocus analysis, *N. azzae* (strain Shy33) forms a distinct, well-supported clade within the family *Zalariaceae*, yet it is clearly separated from known *Zalaria* species. These results support the recognition of *Nigromyces* as a novel genus within the order *Dothideales*, represented by a single species, *N. azzae*.

## Description of *Nigromyces* Al-Sammar H, Boekhout T, Al-Mutairi N, Al-Zarban S gen. nov.

**MycoBank ID**: MB857048

**Etymology**: *Nigromyces* (Ni.gro.my’ces. L. masc. adj. *niger*, black; Gr. masc. n. *mykes*, fungus, mushroom; N.L. masc. n. *Nigromyces*, a black fungus)

**Diagnosis**: Macroscopically, *Nigromyces* differs from *Zalaria* by forming dark-pigmented colonies characterized by prominent meristematic growth, which is more frequent than yeast-like growth, particularly at 30°C. Microscopically, *Nigromyces* lacks the transverse and longitudinal hyphae typically observed in *Zalaria*; instead, it produces hyphae that fragment into arthroconidia. Multilocus phylogenetic analysis based on the ITS region and SSU and LSU rDNA discriminated *Nigromyces* from *Z. alba* and *Z. obscura* (Fig. 1).

**Type species**: *Nigromyces azzae* Al-Sammar H, Boekhout T, Al-Mutairi N, Al-Zarban S

**Description**: The colonies are predominantly black to dark olive brown and meristematic and appear as wrinkled clumps. In some cases, colonies appear moist, with light olive to yellowish pigmentation. This fungus is dimorphic and exhibits restricted meristematic growth along yeast-like phases. Microscopically, it displays hyphal growth with apparent chlamydospore-like structures and budding yeast cells under certain conditions.

## Description of *Nigromyces azzae* AL-Sammar H, Boekhout T, AL-Mutairi N, AL-Zarban S sp. nov.

**MycoBank ID:** MB857050

**Etymology**: *azzae* (az’zae. N.L. gen. n. *azzae*, named in honour of Dr. Azza Al-Musallam, a distinguished mycologist at Kuwait University, for her invaluable contributions to fungal ecology research in Kuwait)

**Diagnosis**: As given above for *Nigromyces*.

**Fig. 2.**
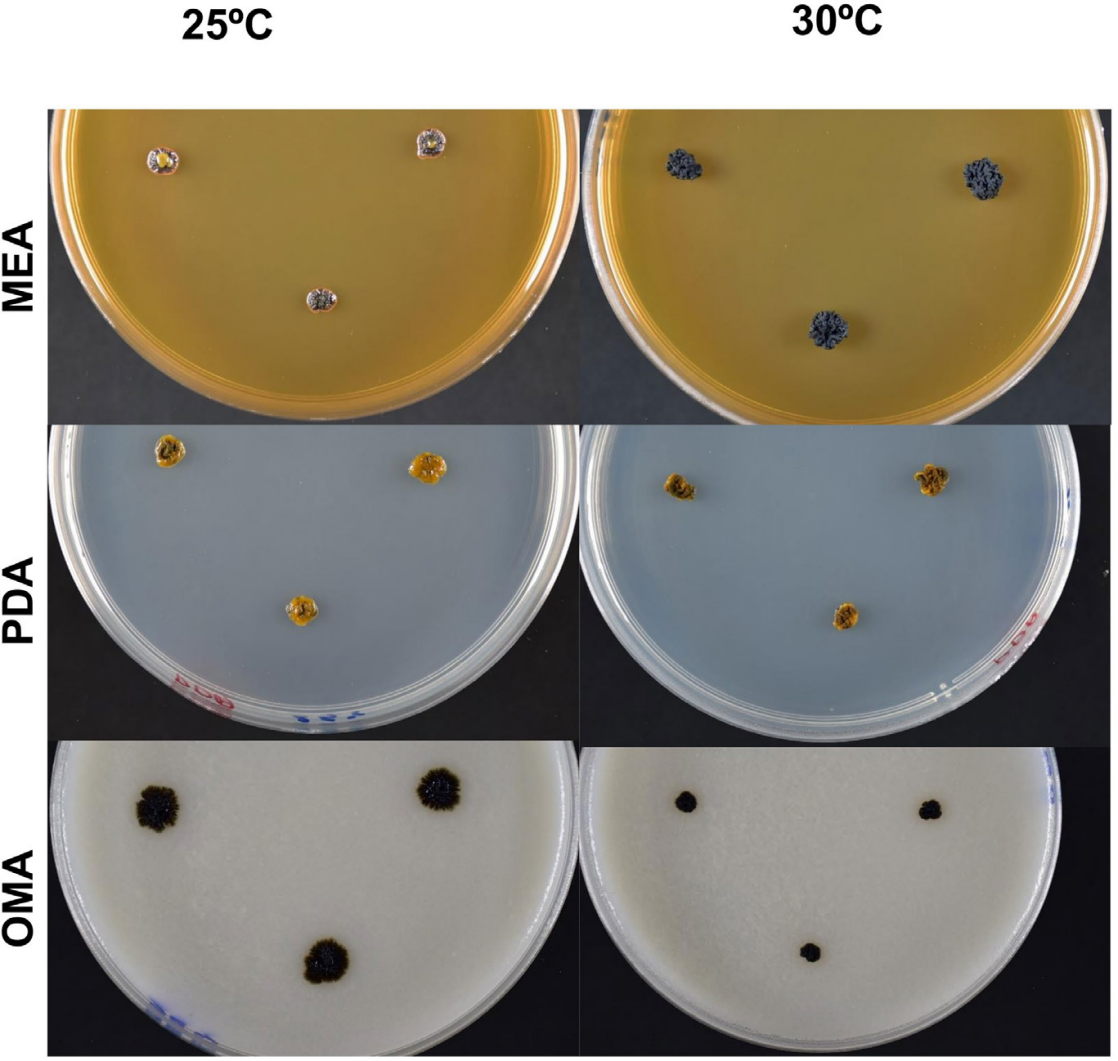
Colony morphology of *N. azzae* CBS 148914^T^ grown on different media and incubation temperatures. Colonies were cultured on MEA, PDA and OMA, and incubated at 25°C (left panel) and 30°C (right panel). Images were taken after 10 days of incubation. At 25°C, colonies on MEA and PDA are wrinkled with glistering surface. At 30°C, colonies on MEA and OMA exhibited meristematic growth with increased pigmentation and compactness.

**Description**: Colonies after 7 days of incubation on malt extract agar (MEA) at 25°C are 20 mm in width, film-like brittle, strongly transversely ridged and folded, glabrous, dull, blackish, lobed entire margin and more olivaceous, with smaller meristematic colonies (Fig. 2). On glucose–yeast extract–peptone agar (GYPA) and DG18 agar, colonies were similar but smaller and pulvinate. Colonies at 30°C on MEA are dark olive exhibiting highly meristematic growth, characterized by clumped growth away from the agar surface (Fig. 2). Colonies grown on Potato Dextrose Agar (PDA) at 25°C light olive to yellowish in colour, wrinkled with glistering surfaces. At a higher temperature of 30°C, colonies are brown to olive-green in colour, showing meristematic growth with rough, dry surface. On oatmeal agar (OMA) at 25°C, colonies are dark brown, whereas edges are lighter brown to olive in colour, surfaces are flat and margin is filamentous. Colony morphology on OMA at 30°C is similar to that at 25°C; however, growth is restricted.

**Fig. 3.**
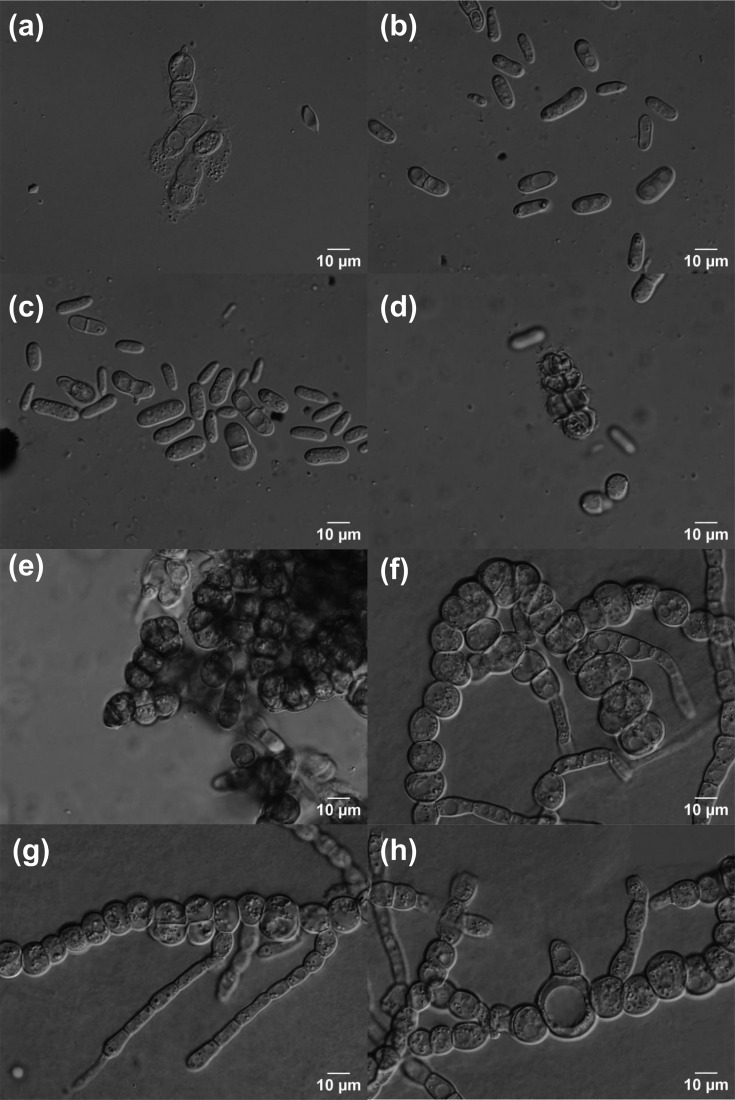
Microscopic images of *N. azzae* CBS 148914**^t^**. (a) Cells in glucose fermentation broth with a single septum. (b, c) Yeast-like cells on MEA, curved to bean-shaped, reproducing by multilateral budding, sometimes on short sterigmata-like outgrowths. (**d)** Blastoconidia and muriform cell clusters with blackish incrusted walls. (**e)** Meristematic clumps formed by endosporulation. (f, g) Arthroconidia formed by hyphal fragmentation on Dalmau plates. (**h)** Inflated, thick-walled hyphal cells with a chlamydospore-like structure.

Yeast cells in glucose fermentation broth are ellipsoidal to dumbbell-shaped, 10–22×4–12 µm, and become 1–3-septate and muriform with age (Fig. 3a). On MEA, cells are 9–21×4–8 µm, curved to bean-shaped, 0–1-septate, with an elongated apex, and reproduce by multilateral budding. Some of the yeast-like cells bud on a short sterigmata-like outgrowth (Fig. 3b, c). Blastoconidia measure 7–12×7–9 µm, small clusters of muriform cells with individual cells measuring 7–13×7–12 µm, cell walls becoming blackish pigmented (Fig. 3d). Meristematic clumps of cells are observed, originating through endosporulation and forming compact, multicellular aggregates (Fig. 3e). On Dalmau plates, hyphae fragment into chains of globose cells/arthroconidia, measuring ~8–22×4–10 µm. Fragmentation occurs by disarticulation at septa, producing short chains or individual arthroconidial cells rather than differentiated conidiophores (Fig. 3f, g). In addition, short chains of inflated, thick-walled hyphal cells are observed measuring 12–23 µm in diameter, that are interpreted as chlamydospore-like structures (Fig. 3h). No sexual morphs were observed on yeast morphology agar, cornmeal agar, GYPA, MEA, 1/10 YM agar, Fowell acetate agar, V8-agar and McLarry agar after up to 2 months of observation.

No fermentation was observed, whereas *N. azzae* assimilated a broad range of carbon sources (Table S1). The species was able to assimilate d-glucose, d-xylose, l-arabinose, sucrose, trehalose, cellobiose, salicin, raffinose, melibiose, soluble starch, meso-erythritol, d-glucitol, d-mannitol and d-glucuronate. Weak growth was observed for d-galactose, d-ribose and d-arabinose, whereas growth was delayed and weak (d, w) for l-sorbose and propane-1,2-diol. Delayed growth was also recorded on l-rhamnose, maltose, methyl α-glucoside, arbutin, ribitol, xylitol, l-arabinitol, d-galacturonate, succinate and ethanol. The *N. azzae* was unable to assimilate d-glucosamine, melibiose, lactose, inulin, glycerol, galactitol, myo-inositol, glucono d-lactone, 2-keto-d-gluconate, d-gluconate, dl-lactate, citrate, methanol, butane-2,3-diol, quinic acid, saccharate or galactonic acid. No growth was observed in the presence of 0.01% or 0.1% cycloheximide or 50% glucose. Starch-like compounds were not produced, and both urease activity and the reaction with diazonium blue B were negative. Growth occurred from 21°C to 42°C, whereas no growth was observed at 18°C and 45°C (Table S1), thus showing thermotolerance. Nitrogen assimilation was assessed using the auxanographic method; however, this approach did not allow for a critical evaluation of nitrogen requirements because of the inconsistent growth patterns of the species.

The GenBank accession numbers for the D1/D2 domain of the LSU are PV018293–PV018308 and OR673877. The accession numbers are OR648401 for the ITS region, OR672588 for the SSU rDNA, OR673877 for the LSU rDNA and PQ782245 for the *TUB2* gene.

**Holotype**: Strain Shy33 is preserved in a metabolically inactive state at the Westerdijk Fungal Biodiversity Institute, Utrecht, the Netherlands, as CBS 148914^T^. It was isolated from a dry leaf of the mangrove tree *A. marina* located in Shuwaikh, Kuwait, in September 2021. The ex-type strain is preserved in the Belgian Coordinated Collections of Microorganisms (Belgium), as MUCL 58501. The MycoBank accession numbers for *Nigromyces* gen. nov. and *N. azzae* sp. nov. are MB857048 and MB857050, respectively. The GenBank accession number for the D1/D2 domain of the LSU is OR673877. The accession numbers are OR648401 for the ITS region, OR672588 for the SSU rDNA, OR673877 for the LSU rDNA and PQ782245 for the *TUB2* gene.

## Conclusion

In the present study, *N. azzae* is identified as a black yeast-like fungus based on its dark-pigmented colonies and prominent meristematic growth. Multilocus phylogenetic analysis based on ITS, SSU and LSU rDNA sequences confirmed that *N. azzae* forms a distinct and well-supported lineage within the family *Zalariaceae*, justifying its recognition as a novel species and the establishment of a new genus. The recovery of a single strain may be attributed to its inherently slow growth rate, which likely led to its exclusion or overgrowth by faster-growing fungi during isolation. Therefore, *N. azzae* can be considered a part of the black yeast-like fungal community inhabiting mangrove ecosystems, and further investigation is needed to explore the ecological distribution and diversity of this novel species.

## Supplementary material

10.1099/ijsem.0.007128 Uncited Supplementary Material 1.

## Abbreviations

BPP: Bayesian posterior probability
GYPA: glucose–yeast extract–peptone agar
MEA: malt extract agar
ML: maximum likelihood
OMA: oatmeal agar
PDA: potato dextrose agar
SDA: Sabouraud Dextrose Agar
UAE: United Arab Emirates
YM: yeast–malt
YPDA: yeast extract peptone dextrose agar
