# Supplementary material for: Nigromyces azzae gen. nov., sp. nov.: a novel black yeast isolated from a mangrove tree in Kuwait
Source: Int J Syst Evol Microbiol. 2026 Apr 10;76(4):007128. doi: 10.1099/ijsem.0.007128 (PMC13068291; doi:10.1099/ijsem.0.007128)
Supplement: Uncited Supplementary Material 1. [file ijsem-76-07128-s001.pdf]

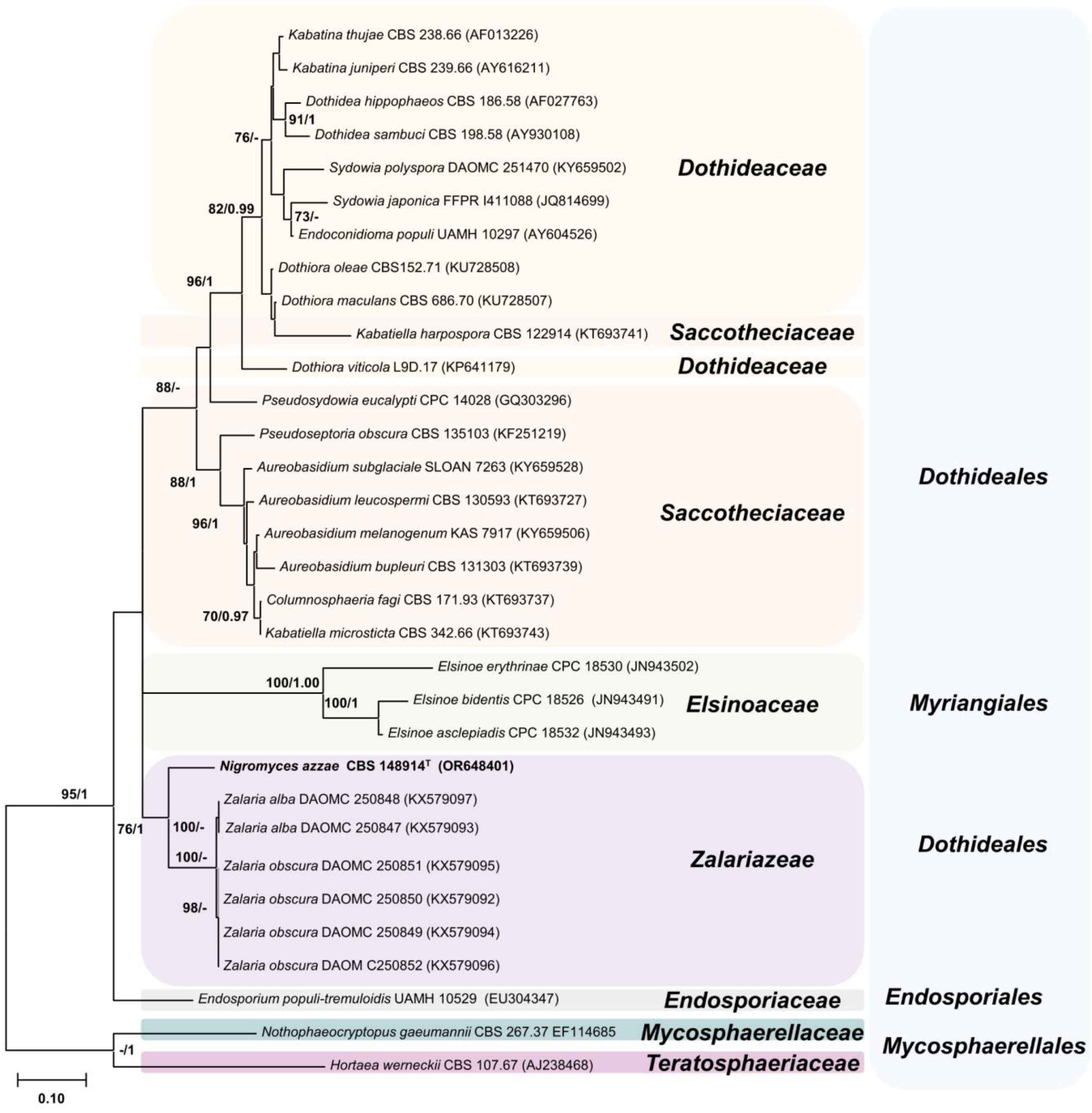

Figure S1. Maximum likelihood phylogenetic tree based on the ITS region of *Nigromyces azzae* sp. nov. and closely related taxa of the *Dothideomycetes*. Species names are followed by their culture collection strain numbers, with GenBank accession numbers in parentheses. Numbers at the nodes represent ML bootstrap support values (BS ≥ 70%, 1000 replicates) followed by BPP values (≥ 95). A dash (“–”) indicates values below the threshold. *Nothophaeocryptopus gaeumannii* was included as outgroup taxa. The scale bar indicates the number of nucleotide substitutions per site.

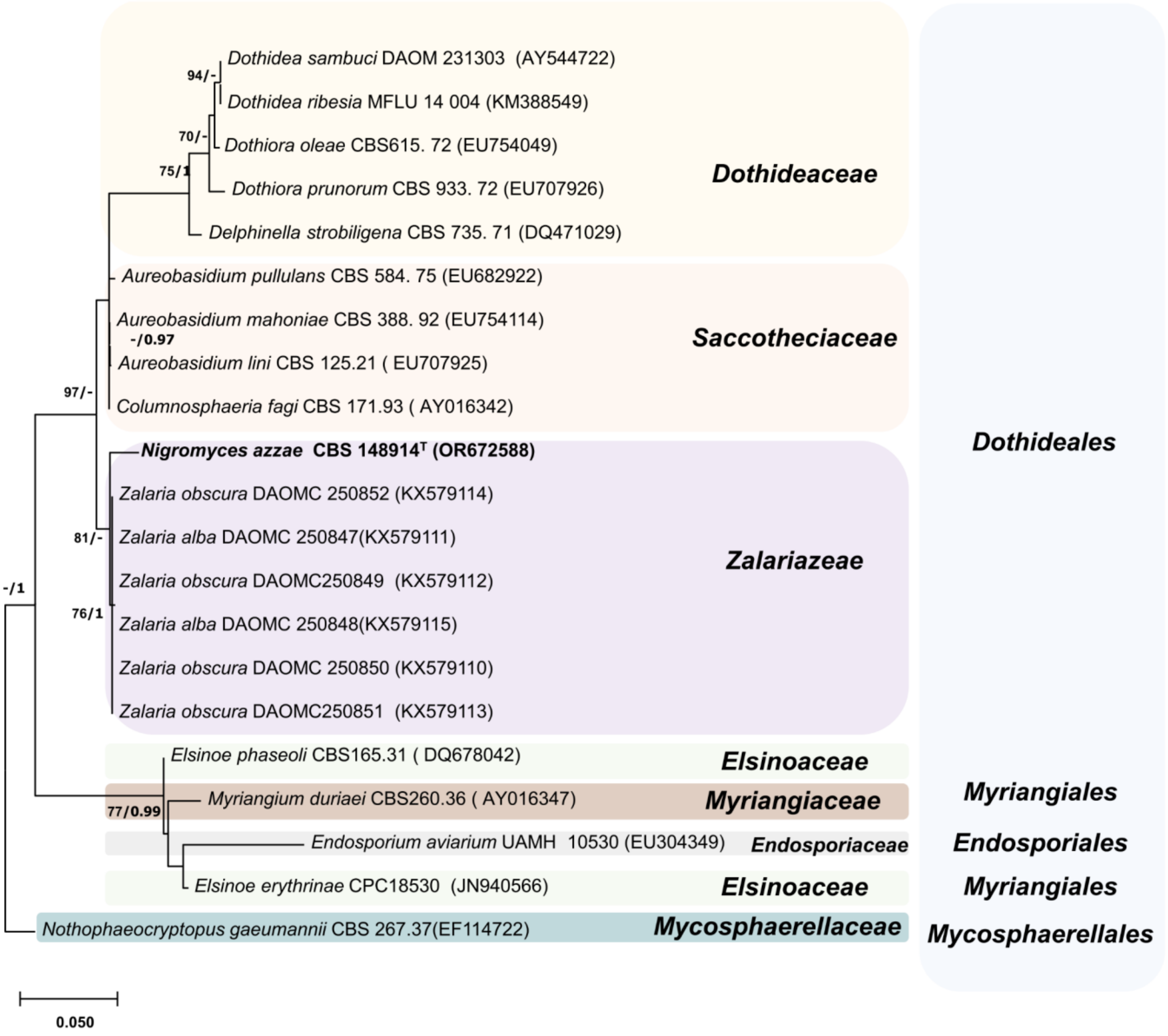

Figure S2. Maximum likelihood phylogenetic tree based on the SSU rDNA region of *Nigromyces azzae* sp. nov. and closely related taxa of the *Dothideomycetes*. Species names are followed by their culture collection strain numbers, with GenBank accession numbers in parentheses. Numbers at the nodes represent ML bootstrap support values (BS ≥ 70%, 1000 replicates) followed by BPP values (≥ 95). A dash ("–") indicates values below the threshold. *Nothophaeocryptopus gaeumannii* was included as an outgroup taxa. The scale bar indicates the number of nucleotide substitutions per site.

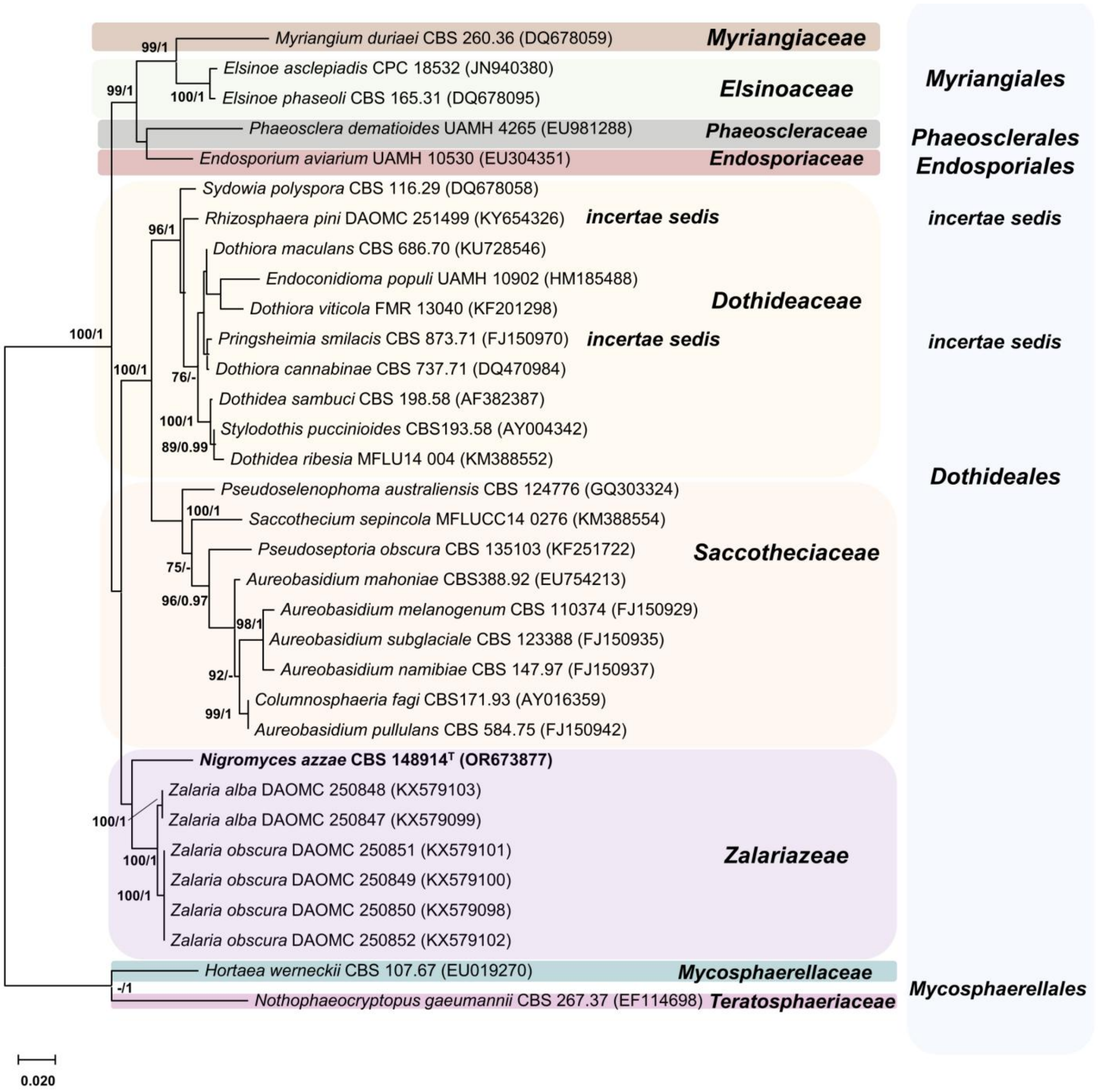

Figure S3. Maximum likelihood phylogenetic tree based on the LSU rDNA region of *Nigromyces azzae* sp. nov. and closely related taxa of the *Dothideomycetes*. Species names are followed by their culture collection strain numbers, with GenBank accession numbers in parentheses. Numbers at the nodes represent ML bootstrap support values (BS ≥ 70%, 1000 replicates) followed by BPP values (≥ 95). A dash ("–") indicates values below the threshold. *Hortaea werneckii* and *Nothophaeocryptopus gaeumannii* was included as an outgroup taxa. The scale bar indicates the number of nucleotide substitutions per site.

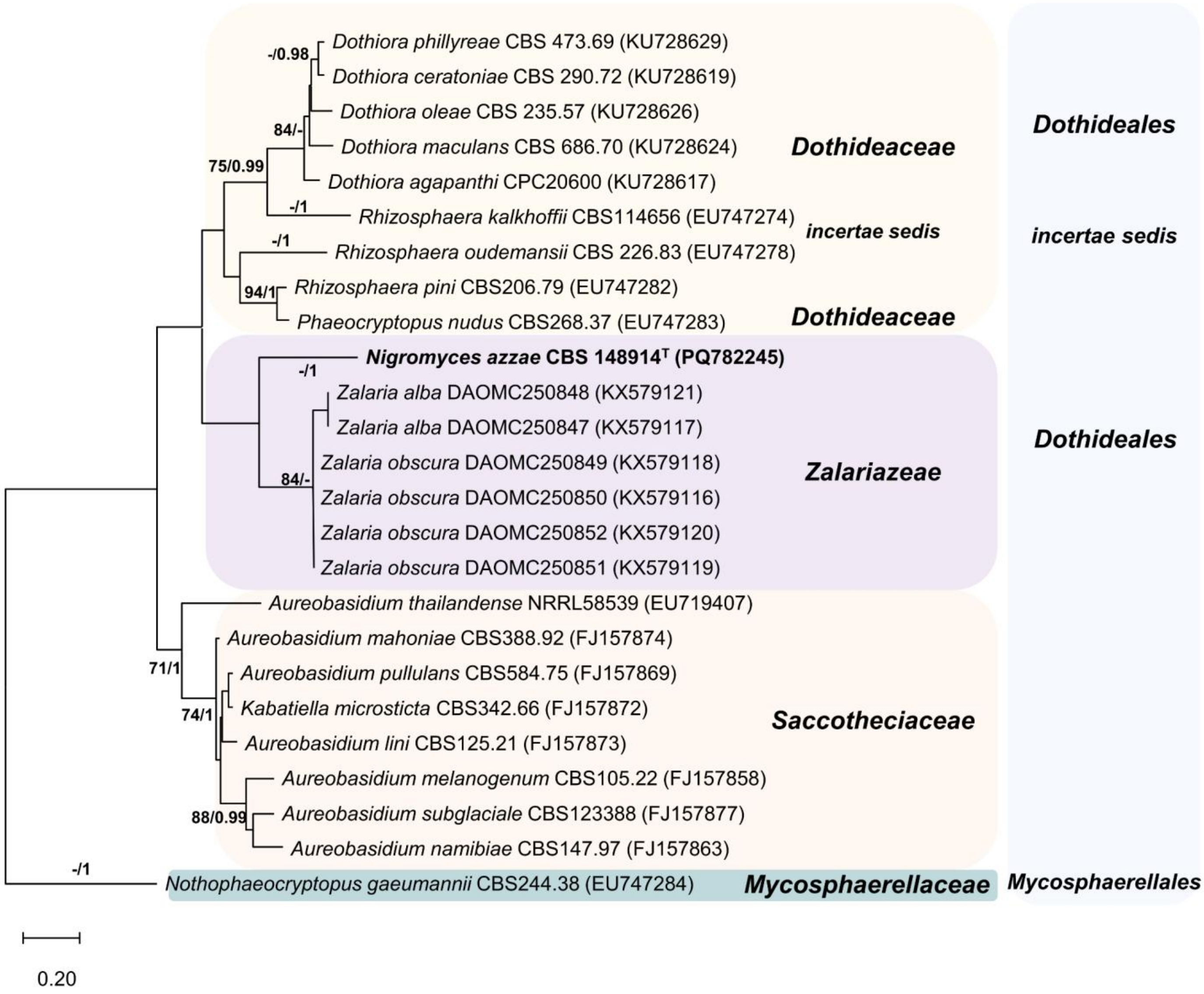

Figure S4. Maximum likelihood phylogenetic tree based on the *TUB2* gene of *Nigromyces azzae* sp. nov. and closely related taxa of the *Dothideomycetes*. Species names are followed by their culture collection strain numbers, with GenBank accession numbers in parentheses. Numbers at the nodes represent ML bootstrap support values (BS ≥ 70%, 1000 replicates) followed by BPP values (≥ 95). A dash ("–") indicates values below the threshold. *Nothophaeocryptopus gaeumannii* was included as an outgroup taxa. The scale bar indicates the number of nucleotide substitutions per site.

Table S1. Physiological characteristics of *N. azzae*

**Assimilation of carbon compounds**	***N. azzae***
D-glucose	+
D-galactose	w
L-sorbose	d,w
D-glucosamine	-
D-ribose	w
D-xylose	+
L-arabinose	+
D-arabinose	w
L-rhamnose	d
Sucrose	+
Maltose	d
α,α trehalose	+
Methyl α-glucoside	d
Cellobiose	+
Salicin	+
Arbutin	d
Melibiose	-
Lactose	-
Raffinose	+
Melezitose	+
Inuline	-
Soluble starch	+
Glycerol	-
Meso erythritol	+
Ribitol	d
Xylitol	d
L-arabinitol	d
D-glucitol	+
D-mannitol	+
Galactitol	-
Myo-inositol	-
Glucono d-lactone	-
2-keto-D-gluconate	-
D-gluconate	-
D-glucuronate	+
D-galacturonate	d
DL-lactate	-
Succinate	d
Citrate	-

Table S1. Continued.

**Assimilation of carbon compounds**	***N. azzae***
Methanol	-
Ethanol	d
Propane 1,2 diol	d,w
Butane 2,3 diol	-
Quinic acid	-
Saccharate	-
Galactonic acid	-
**Other tests**	
Growth with 0.01% cycloheximide	-
Growth with 0.1% cycloheximide	-
50% glucose	-
60% glucose	-
Starch production	-
Urea test	-
DBB reaction	-
**Growth at various temperatures**	
18 °C	-
21 °C	+
25 °C	+
30 °C	+
35 °C	+
37 °C	+
40 °C	+
42 °C	+
45°C	-

Growth test results: +, positive, -, negative, w, weak, d, delayed
